# Surgical Anatomy and Prevalence of Intracranial Aneurysms in Patients With Spontaneous Subarachnoid Hemorrhage

**DOI:** 10.7759/cureus.20463

**Published:** 2021-12-16

**Authors:** Sahibzada Haseeb Ahmed, Muhammad Haris, Najma Baseer, Aqsa Saleema, Sobia Haris, Farah Deeba, Muhammad Jehangir Khan

**Affiliations:** 1 Department of Neurosurgery, Police Services Hospital, Peshawar, PAK; 2 Department of Anatomy, Nowshera Medical College, Nowshera, PAK; 3 Department of Anatomy, Institute of Basic Medical Sciences, Khyber Medical University, Peshawar, PAK; 4 Department of Anatomy, Jinnah Medical College, Peshawar, PAK; 5 Department of Medical Education, Nowshera Medical College, Nowshera, PAK; 6 Department of Pediatric Surgery, Makka Medical Center, Nowshera, PAK

**Keywords:** prevalence, surgical anatomy, spontaneous subarachnoid hemorrhage, intracranial aneurysm, cerebral aneurism, anatomy

## Abstract

Objective

To assess the surgical anatomy and prevalence of intracranial aneurysms in patients with spontaneous subarachnoid hemorrhage.

Materials and methods

The current research investigated a total of 119 individuals from Peshawar, Pakistan. All the adult patients in the age range of 30 to 60 years, of both genders, presenting with spontaneous subarachnoid hemorrhage were included. A thorough history was taken, as well as a full, general, physical, systemic, and neurological examination was done. All individuals who arrived with a rapid onset of severe headache, with or without loss of consciousness, and had a CT scan showing spontaneous subarachnoid hemorrhage were included. All patients were subjected to CT angiography in the hospital to ascertain any intracranial aneurysm. CT angiography was done by a consultant radiologist (FCPS) having at least five years of experience. All the above-mentioned information, including age, diabetes, obesity, smoking, gender, and hypertension, was recorded in a predesigned proforma.

Results

The current study found that among 119 patients, 24 (20%) were of age 30-40 years, 44 (37%) were between 41 and 50 years, and 51 (43%) were of age 51 to 60 years; 73 (61%) were male and 46 (39%) were female; 67 (56%) were obese and 52 (44%) were not obese; 81 (68%) patients were hypertensive and 38 (32%) patients were non-hypertensive; 75 (63%) patients were diabetic and 44 (37%) patients were non-diabetic; 49 (41%) patients were smokers and 70 (59%) patients were non-smokers. Moreover, 96 (81%) patients had intracranial aneurysms and 23 (19%) patients did not have intracranial aneurysms.

Conclusion

As has been observed, the prevalence of intracranial aneurysms and surgical anatomy was 81% in individuals from Peshawar, Pakistan, with spontaneous subarachnoid hemorrhage.

## Introduction

Besides trauma, the most prevalent cause of subarachnoid hemorrhage (SAH) with a fatal consequence is a burst cerebral aneurysm. The kind of bleeding is either spontaneous (non-traumatic) or traumatic. Spontaneous subarachnoid hemorrhage (SAH) occurs as a result of the rupture of an aneurysm or arterio-venous malformation, or as a result of hypertension or an unexplained cause. They account for 5% of all strokes and are half as common as intraparenchymal hemorrhages [1-2]. The most prevalent cause of spontaneous SAH is a ruptured intracranial aneurysm (approximately 85% of cases) [3]. SAH is a devastating condition with a 50% death rate and one-third of survivors being functionally dependent [4]. The global frequency of spontaneous subarachnoid hemorrhage is around 9.1/100,000 people a year, however, there is a lack of data on SAH incidence and prognosis in African-American communities in America [5]. The prevalence of SAH in Sub-Saharan Africa is unclear, and there have been few investigations on SAH in African populations. The frequency of stroke due to subarachnoid hemorrhage (SAH) stays stable, with intracranial aneurysm rupture producing SAH in up to 5000 individuals in the United Kingdom each year [6]. Although this accounts for fewer than 5% of all strokes, detection is critical since action can drastically change the outcome. Aneurysm rupture has a combined death and morbidity rate of 50%; because the disorder can strike anyone of any age, long-term morbidity in survivors can be significant [7]. Failure to identify SAH exposes a patient to the potentially deadly consequences of another bleed, as well as problems that may be prevented or properly managed [8]. However, the physiology of aneurysm growth and rupture is poorly studied, and epidemiological research on SAH might aid us in future studies on the disease's natural history. It can also aid in the identification of risk and prognosis variables that may lead to disease mechanism indicators. Furthermore, there is a general dearth of knowledge on SAH occurrence and prognosis in several regions of the world, notably among people of African descent [6]. Anterior cerebral circulation has been observed to be affected in approximately 96% of patients, with the posterior cerebral circulation affecting only 3.4%. Furthermore, between 3% and 15% of patients had numerous aneurysms [7-8]. In one study, the prevalence of intracranial aneurysms was 72.8% in patients with spontaneous subarachnoid hemorrhage [9]. In other studies, the prevalence of intracranial aneurysms was 79.3% and 81% in patients with spontaneous subarachnoid hemorrhage respectively [6,10]. In another study, the frequency of intracranial aneurysms was as high as 88.9% in individuals having spontaneous subarachnoid hemorrhage [11]. Thus, this study was aimed to assess the presence or absence, as well as surgical anatomy of intracranial aneurysms in patients with spontaneous subarachnoid hemorrhage. As a result, no similar study has been undertaken at our facility in the previous three years, therefore this study will provide us with the most recent statistics to the extent of prevalence of cerebral aneurysms and surgical anatomy in patients with spontaneous subarachnoid hemorrhage in our facility.

## Materials and methods

Study design and setting

This was descriptive cross-sectional research conducted at Lady Reading Hospital at the Department of Neurosurgery in Peshawar, Pakistan.

Study duration, sample size, and sampling technique

This study was done from 14/03/2020 to 13/09/2020 after receiving ethical permission from the Institutional Ethical Review Board (IERB) of Nowshera Medical College, Nowshera, Pakistan, via letter No: 27/NMC/IERB/Sec dated 10/03/2020. The sample size was obtained using the WHO formula for sample size calculation by taking 72.8% of the total population [9], the prevalence of intracranial aneurysms and surgical anatomy in patients with spontaneous subarachnoid hemorrhage, confidence interval (CI) is 95%, and margin of error 5% with a p-value of ≤0.05 considered as substantial. In the analysis, the non-probability consecutive sampling approach was used.

Sample selection

This research covered all adult individuals between the ages of 30 and 60, of either gender, who presented with spontaneous subarachnoid hemorrhage. Patients with a history of any trauma, patients with bleeding disorders (assessed on the history and laboratory investigations), and patients with deranged liver functions and renal functions (assessed on the clinical examination and laboratory investigations) were excluded.

Data collection

All individuals who met the inclusion criteria, i.e., those who presented with spontaneous subarachnoid hemorrhage, were enrolled in the research through the Lady Reading Hospital's OPD/Emergency and Neurosurgery departments in Peshawar. The study's goal and benefits were described to the patients who agreed to participate, and formal informed consent was acquired. A thorough history was taken, as well as a full general physical, systemic, and neurological examination. All individuals who presented with sudden onset of severe headache with or without loss of consciousness having spontaneous subarachnoid hemorrhage were subjected to CT-angiography in the hospital to ascertain the presence of an intracranial aneurysm. CT-angiography was done by a consultant radiologist (FCPS) having at least five years of experience. All the above-mentioned information, including age, diabetes, obesity, smoking, gender, and hypertension, was recorded in a predesigned proforma.

Data analysis

The acquired data were entered into SPSS version 23 (IBM Corp., Armonk, NY) for descriptive analysis, and descriptive statistical tests were run. For quantitative factors, such as age, mean and standard deviation, were calculated. For categorical factors, such as gender, obesity, hypertension, diabetes, smoking, and intracranial aneurysms, frequency and percentages were calculated. To test if there was any impact modification, intracranial aneurysms were stratified by age, gender, obesity, hypertension, diabetes, and smoking. The post-stratification chi-square test was employed, with a p-value of ≤0.05 deemed significant.

## Results

The current study found that among 119 patients, 24 (20%) were aged 30 to 40 years, 44 (37%) were 41 to 50 years, and 51 (43%) were aged 51 to 60 years; 73 (61%) were male and 46 (39%) were female; 67 (56%) were obese and 52 (44%) were not obese; 81 (68%) patients were hypertensive and 38 (32%) patients were non-hypertensive, 75 (63%) patients were diabetic and 44 (37%) patients were non-diabetic; 49 (41%) patients were smokers and 70 (59%) patients were nonsmokers. Moreover, 96 (81%) patients had intracranial aneurysms and 23 (19%) patients did not have intracranial aneurysms. Stratification of intracranial aneurysms with respect to age, gender, obesity, hypertension, diabetes, and smoking is shown in Tables 1-6.

**Table 1:** Stratification of Intracranial Aneurysms With Respect to Age Distribution

Intracranial aneurysms	30-40 years	41-50 years	51-60 years	Total	p-value
Yes	19	36	41	96	0.9634
No	5	8	10	23	
Total	24	44	51	119	

**Table 2:** Stratification of Intracranial Aneurysms With Respect to Gender Distribution

Intracranial aneurysms	Male	Female	Total	p-value
Yes	59	37	96	0.9584
No	14	9	23	
Total	73	46	119	

**Table 3:** Stratification of Intracranial Aneurysms With Respect to Obesity

Intracranial aneurysms	Obese	Non-obese	Total	p-value
Yes	54	42	96	0.9811
No	13	10	23	
Total	67	52	119	

**Table 4:** Stratification of Intracranial Aneurysms With Respect to Hypertension

Intracranial aneurysms	Hypertensive	Non-hypertensive	Total	p-value
Yes	65	31	96	0.8637
No	16	7	23	
Total	81	38	119	

**Table 5:** Stratification of Intracranial Aneurysms With Respect to Diabetes Mellitus

Intracranial aneurysms	Diabetic	Non-diabetic	Total	p-value
Yes	60	36	96	0.8084
No	15	8	23	
Total	75	44	119	

**Table 6:** Stratification of Intracranial Aneurysms With Respect to Smoking

Intracranial aneurysms	Yes	No	Total	p-value
Yes	39	57	96	0.8027
No	10	13	23	
Total	49	70	119	

## Discussion

The current study found that among 119 patients, 24 (20%) were aged 30 to 40 years, 44 (37%) were 41 to 50 years, and 51 (43%) were aged 51 to 60 years; 73 (61%) were male and 46 (39%) were female; 67 (56%) were obese and 52 (44%) patients were non-obese, 81 (68%) patients were hypertensive and 38 (32%) patients were non-hypertensive, 75 (63%) patients were diabetic and 44 (37%) patients were non-diabetic; 49 (41%) patients were smokers and 70 (59%) patients were non-smokers. Moreover, 96 (81%) patients had intracranial aneurysms and 23 (19%) individuals did not have intracranial aneurysms. Comparable findings were found by Schertz M et al. [12]: 121 individuals suffered a SAH throughout the research duration, with a huge prevalence of female cases (71.1% versus 28.9%, p<0.001) and an average age of 57.1 years. In 96 SAH instances, the cause was determined to be aneurismal (79.3%). The yearly crude incidence was 4.36 per 100,000 people (CI 95%, 2.30-6.42). The global age-standardized occurrence was 3.29/100,000 people a year (CI 95% 1.74-4.84). Twenty-nine patients passed away within 30 days of being diagnosed with SAH (case death rate: 24% (CI 95%, 16.4-31.6)).

Similar findings were seen in another research conducted by Ronne-Engström E et al., which included demographic and clinical data from 615 patients with SAH hospitalized between 2007 and 2011 [13]. Aneurysms were discovered in 448 patients (72.8%). They were related to an earlier published series from our center's aneurysm group (n=676) in the years 2001-2006. Linear regression was performed to identify characteristics that predicted practical outcomes in the aneurysm group as a whole (2001-2011).

Song JP et al. conducted another research in which 2562 individuals were included, with 81.4% of them having aneurismal SAH and 18.6% having non-aneurismal SAH [14]. With no fatalities, the total complication frequency of emergent digital subtraction angiography (DSA) was 3.9%. There were 321 cases (15.4%) of multiple aneurysms among patients with aneurismal SAH, and a total of 2435 aneurysms were diagnosed. The anterior communicating artery (30.1%), posterior communicating artery (28.7%), and middle cerebral artery were the most common sites for aneurysms (15.9%), 76.5% (n = 365) of non-aneurismal SAH patients had a negative initial DSA, with 62 cases having peri-mesencephalic non-aneurismal SAH (PNSAH). Repeat DSA or CTA was performed on 252 patients who had a negative initial DSA, including 45 PNSAH cases. The repetitive angiographic findings in 45 PNSAH patients were negative while 28 (13.5%) cerebral aneurysms were found in the other 207 cases. Furthermore, the most common causes of non-aneurismal SAH were brain arteriovenous malformation (AVM, 7.5%), Moyamoya disease (7.3%), stenosis, or sclerosis of the cerebral artery (2.7%), and dural arteriovenous fistula or carotid-cavernous fistula (2.3%). Elhadi AM et al. did another investigation and reported that the prevalence of intracranial aneurysms was as high as 88.9% in patients with spontaneous subarachnoid hemorrhage [15].

More research on diverse populations of SAH patients is needed before any suggestions on the suitability of screening for intracranial aneurysms in SAH individuals can be made.

## Conclusions

As has been observed, the prevalence of intracranial aneurysms and surgical anatomy was 81% in individuals from Peshawar, Pakistan, with spontaneous subarachnoid hemorrhage.
